# Author Correction: Ferromagnetic quasi-atomic electrons in two-dimensional electride

**DOI:** 10.1038/s41467-020-16377-4

**Published:** 2020-05-14

**Authors:** Seung Yong Lee, Jae-Yeol Hwang, Jongho Park, Chandani N. Nandadasa, Younghak Kim, Joonho Bang, Kimoon Lee, Kyu Hyoung Lee, Yunwei Zhang, Yanming Ma, Hideo Hosono, Young Hee Lee, Seong-Gon Kim, Sung Wng Kim

**Affiliations:** 1 Department of Energy Science, Sungkyunkwan University, Suwon, 16419 Republic of Korea; 2 Center for Integrated Nanostructure Physics, Institute for Basic Science, Suwon, 16419 Republic of Korea; 3 Department of Physics, Pukyong National University, Busan, 48513 Republic of Korea; 4 Department of Physics & Astronomy and Center for Computational Sciences, Mississippi State University, Mississippi State Mississippi, 39762 USA; 5 Pohang Accelerator Laboratory, Pohang University of Science and Technology, Pohang, 37673 Republic of Korea; 6 Department of Physics, Kunsan National University, Gunsan, 54150 Republic of Korea; 7 Department of Materials Science and Engineering, Yonsei University, Seoul, 03722 Republic of Korea; 8 State Key Laboratory of Superhard Materials & Innovation Center of Computational Physics Methods and Software, College of Physics, Jilin University, Changchun, 130012 China; 9 Materials Research Center for Element Strategy, Tokyo Institute of Technology, Yokohama, 226-8503 Japan

**Keywords:** Ferromagnetism, Magnetic properties and materials

Correction to: *Nature Communications* 10.1038/s41467-020-15253-5, published online 23 March 2020.

The original version of this Article contained an error in the author affiliations.

The affiliation of Sung Wng Kim with ‘Center for Integrated Nanostructure Physics, Institute for Basic Science, Suwon 16419, Republic of Korea’ was inadvertently omitted.

The original version of this Article omitted the following from the Acknowledgements:

‘Institute for Basic Science (IBS-R011-D1)’

This has now been corrected in both the PDF and HTML versions of the Article.

