# Supplementary material for: Ferromagnetic quasi-atomic electrons in two-dimensional electride
Source: Nat Commun. 2020 Mar 23;11:1526. doi: 10.1038/s41467-020-15253-5 (PMC7090050; doi:10.1038/s41467-020-15253-5)
Supplement: Supplementary file 1 — Supplementary Information [file 41467_2020_15253_MOESM1_ESM.pdf]

Supplementary Information

Ferromagnetic quasi-atomic electrons in two-dimensional electride

Seung Yong Lee et al.

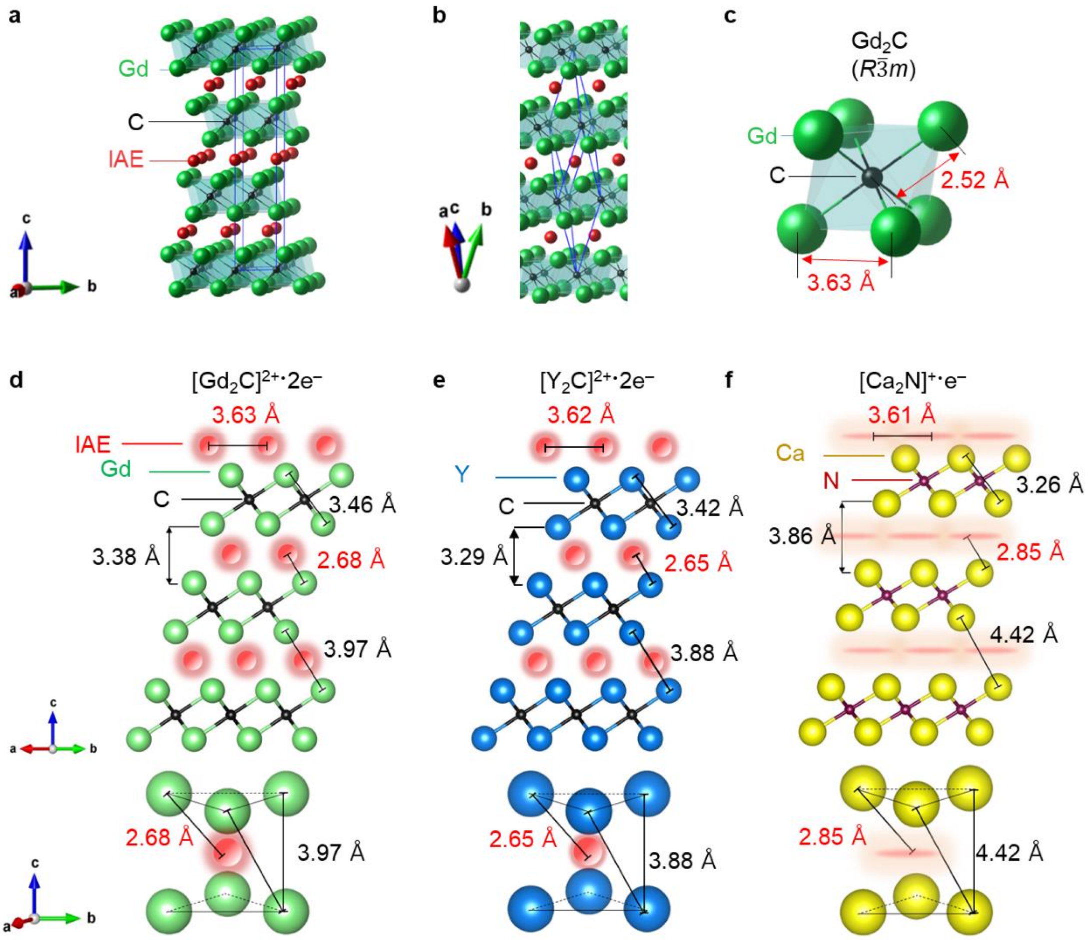

Supplementary Figure 1 Crystal structure of $[Gd_2C]^{2+}\cdot 2e^-$ electride and comparison of structural parameters of 2D electrides. **a** Crystal structure of $[Gd_2C]^{2+}\cdot 2e^-$ electride in hexagonal coordinates. **b** Rhombohedral primitive cell of (**a**). **c** Octahedral Gd_6C sub-structure, representing Gd-Gd and Gd-C bond lengths. **d**–**f** Comparison of structural parameters of $[Gd_2C]^{2+}\cdot 2e^-$ (**d**), $[Y_2C]^{2+}\cdot 2e^-$ (**e**), $[Ca_2N]^{+}\cdot e^-$ (**f**). The interlayer space of $[Gd_2C]^{2+}\cdot 2e^-$ electride (~3.38 Å) is shorter than that of $[Ca_2N]^{+}\cdot e^-$ (~3.86 Å)[S1] and slightly larger than that of $[Y_2C]^{2+}\cdot 2e^-$ (~3.29 Å)[S2]. While a common feature among the three 2D electrides is that the outermost orbital character of neighboring atoms interacting with IAEs is the *d*-orbital of metal cations, a critical difference from the reported 2D electrides should be noted for $[Gd_2C]^{2+}\cdot 2e^-$: the interlayer space (~3.38 Å) is shorter than that of $[Ca_2N]^{+}\cdot e^-$ (~3.86 Å) and similar to that of $[Y_2C]^{2+}\cdot 2e^-$ (~3.29 Å); consequently, the IAEs in $[Gd_2C]^{2+}\cdot 2e^-$ are strongly localized in the interlayer space in contrast to the fully delocalized IAEs in $[Ca_2N]^{+}\cdot e^-$ and similar to those in $[Y_2C]^{2+}\cdot 2e^-$. The interatomic distances of quasi-atomic IAEs in $[Gd_2C]^{2+}\cdot 2e^-$ (~3.63 Å) and $[Y_2C]^{2+}\cdot 2e^-$ (~3.62 Å) are similar to that of IAEs maxima in $[Ca_2N]^{+}\cdot e^-$ (~3.61 Å).

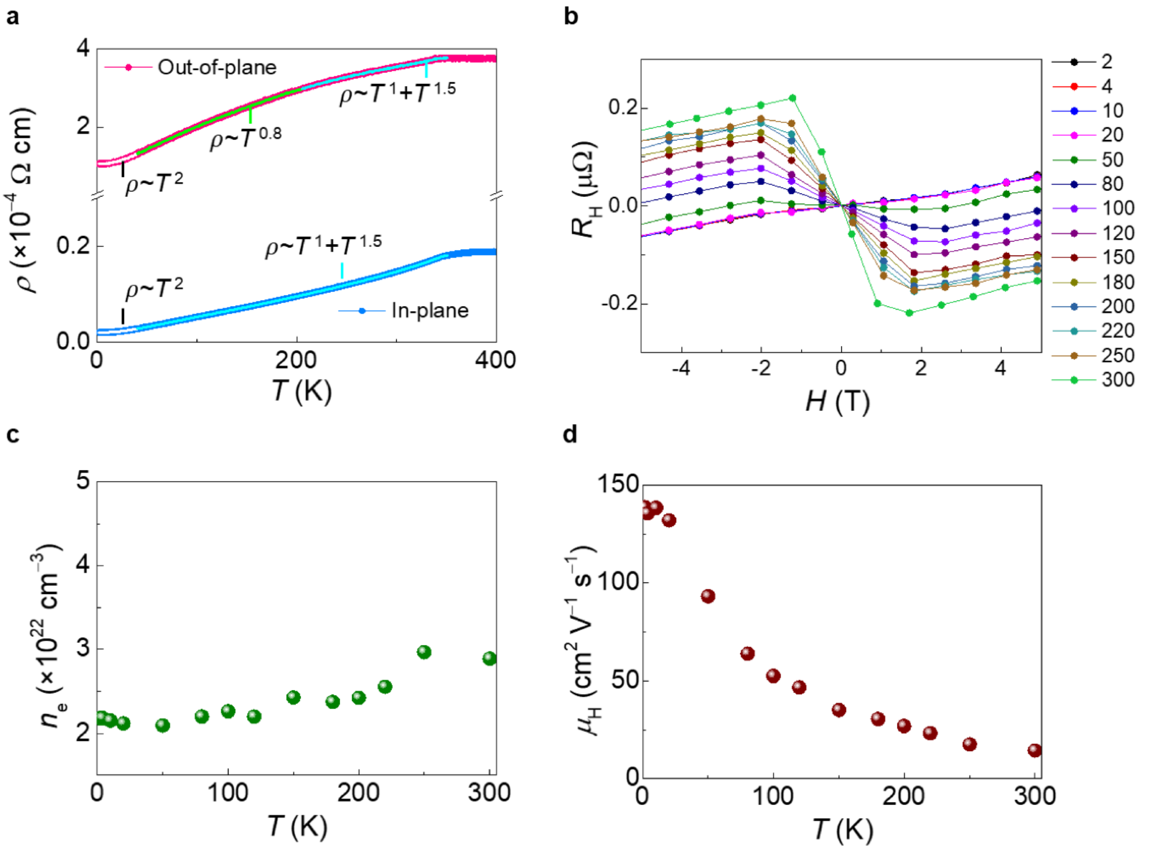

Supplementary Figure 2 Electrical transport properties of $[Gd_2C]^{2+}\cdot 2e^-$ electride. a Temperature dependence of electrical resistivity in the out-of-plane and in-plane directions for cleaved surface of $[Gd_2C]^{2+}\cdot 2e^-$ electride. In the power-law fitting ($\rho(T) = \rho_0 + AT^n$) for the temperature (T) dependence of electrical resistivity (ρ), n values of 0.8 and 1.5 over the range 40 K < T < 350 K were obtained in the out-of-plane and in-plane directions, respectively, indicating that both behaviors are ascribed to the scattering of itinerant electrons with magnetic spins. **b** Magnetic field (H) dependence of Hall coefficient (R_H). **c** Temperature dependence of carrier concentration obtained from (**b**). The carrier concentration (n_e) estimated from the linear H dependence of the Hall effect assuming the free electron model is ~2.9×10^{22} cm^{-3} at 300 K, which is similar to the theoretical n_e of 2.84×10^{22} cm^{-3} based on the chemical formula of $[Gd_2C]^{2+}\cdot 2e^-$. **d** Temperature dependence of carrier mobility (μ_H).

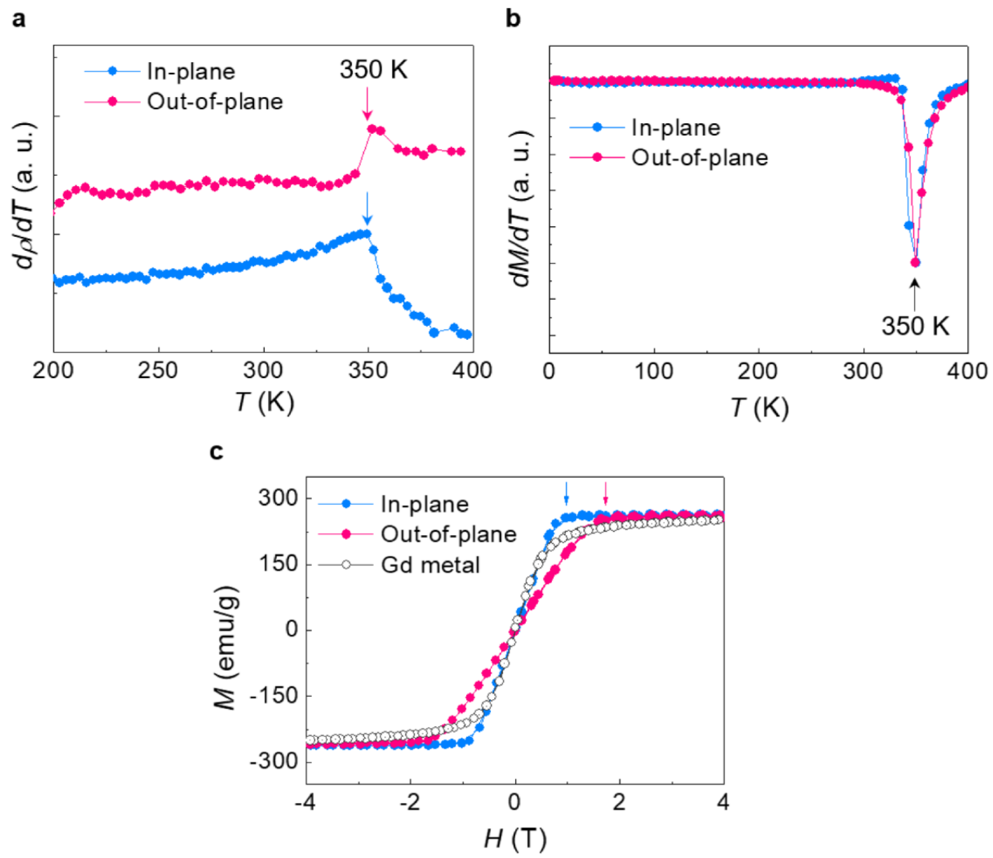

Supplementary Figure 3 Magnetic transition temperature of ferromagnetic $[Gd_2C]^{2+}\cdot 2e^-$ electride. **a** Temperature dependence of $d\rho/dT$ and **b** dM/dT. **c** Magnetic field (H) dependence of magnetization (M) in the in-plane and out-of-plane directions of $[Gd_2C]^{2+}\cdot 2e^-$ electride and Gd metal.

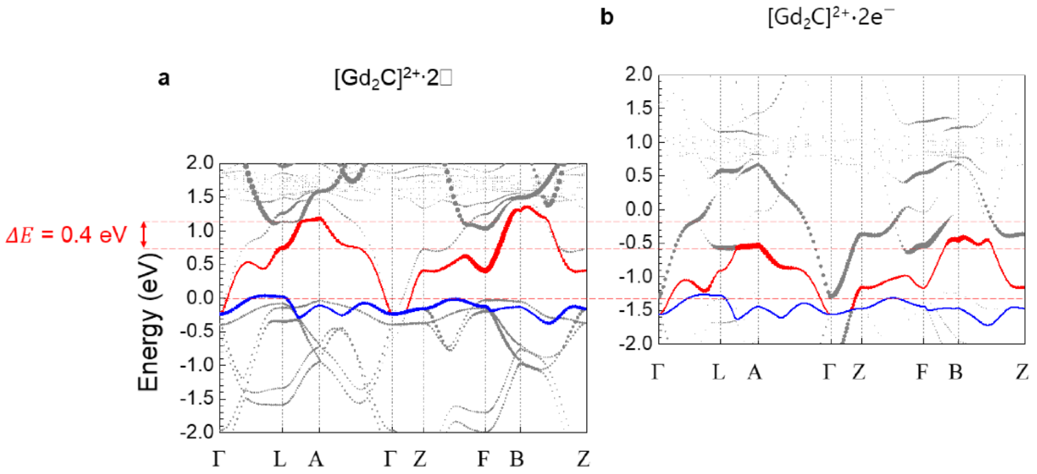

Supplementary Figure 4 Comparison of the orbital energies of the band structure in the $[Gd_2C]^{2+}$·*y*□·(2–*y*)e^- system. □ represents the vacancy of IAE. **a** Gd-*d* orbital of $[Gd_2C]^{2+}$·2□ (blue curves) and **b** IAE-*s* orbital of $[Gd_2C]^{2+}$·2e^- (red curves), which is considered to be formed by adding two electrons to $[Gd_2C]^{2+}$·2□. The two band structures are aligned by matching the bands at the Fermi level of the $[Gd_2C]^{2+}$·2□ system (blue curves). The bands not relevant to the aligning have been grayed out for clarity.

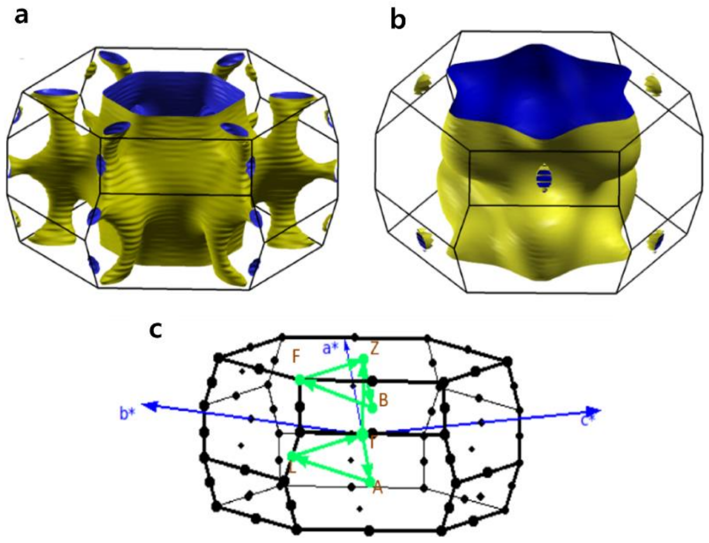

Supplementary Figure 5 Fermi surfaces of $[Gd_2C]^{2+}\cdot 2e^-$ electride. a–b Spin-up (**a**) and spin-down (**b**) Fermi surfaces. **c** High symmetry special *k*-points of the reciprocal lattice.

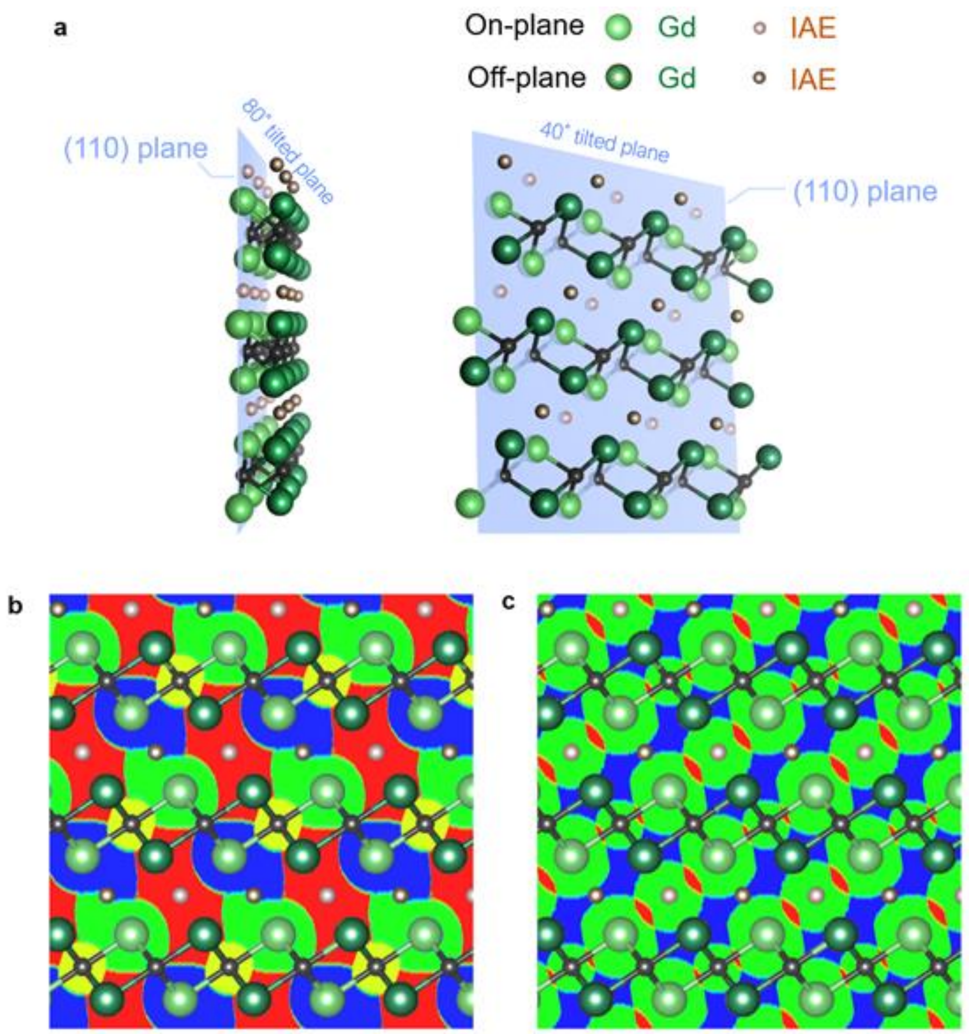

Supplementary Figure 6 Comparison of Bader basin and projection spheres of $[Gd_2C]^{2+}\cdot 2e^{-}$ electride plotted on (110) plane. a Atomic configuration on (110) plane of $[Gd_2C]^{2+}\cdot 2e^{-}$ in different angles. The different color of elements represents the on-plane or off-plane from (110) plane. Light and dark green (brown) balls of Gd atoms (IAEs) are located on the plane and off the plane, respectively. **b** Bader basin for the computation of local magnetic moments of $[Gd_2C]^{2+}\cdot 2e^{-}$ using the Bader decomposition method. There is no volume unaccounted and no overlapped volume. Colors of each element are the same as the colors of atoms and IAEs showed in **a**. **c** Projection spheres are assigned to each atom when the projection method is employed as shown in Supplementary Tables 3 and 4. The volumes of spheres cover 92.5 % of the total volume. There are unaccounted (blue) and double-counted (red) regions.

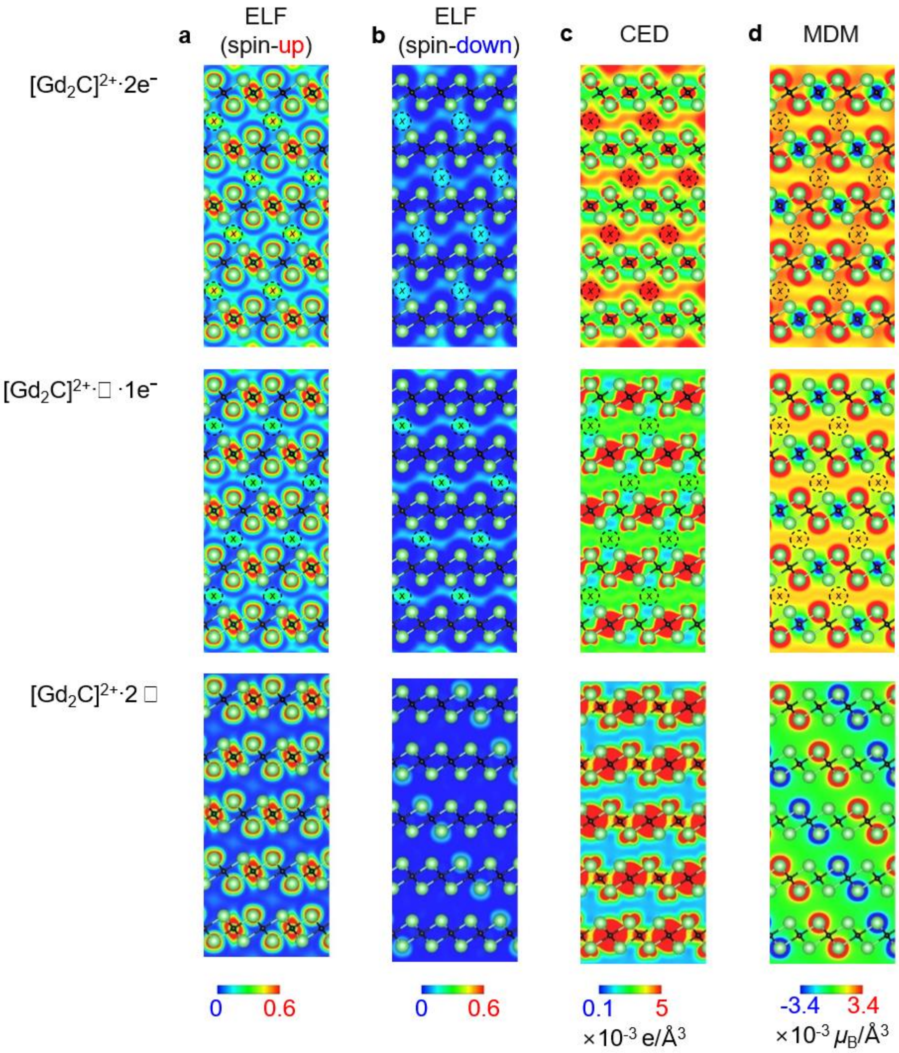

Supplementary Figure 7 Electronic and magnetic structures of the $[Gd_2C]^{2+}$·*y*□·(2–*y*)e^- system. "*X*" in the dashed circle denotes the site of IAEs. **a**–**b** Electron localization function (ELF) for spin-up (**a**) and spin-down (**b**) states. **c** Conduction electron density (CED) map. **d** Magnetization density map (MDM).

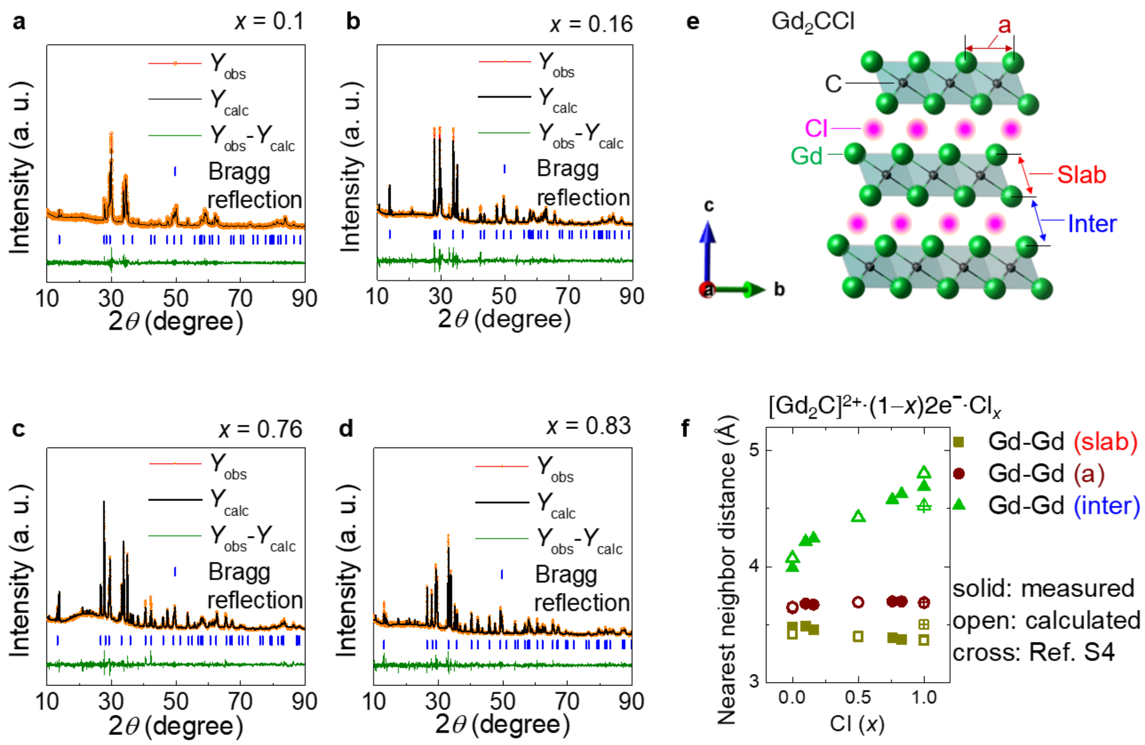

Supplementary Figure 8 Structure refinement of $[Gd_2C]^{2+}\cdot(1-x)2e^{-}\cdot Cl_x$ system. a–d Rietveld refinements of X-ray diffraction patterns for $[Gd_2C]^{2+}\cdot(1-x)2e^{-}\cdot Cl_x$ system. (**a**) x = 0.1, (**b**) x = 0.16, (**c**) x = 0.76, and (**d**) x = 0.83. **e** Schematic illustration of the layered crystal structure of Gd_2CCl. Arrows indicate distances between adjacent Gd atoms in slab and interlayer space. **f** Plot of nearest neighbor distance between Gd atoms.

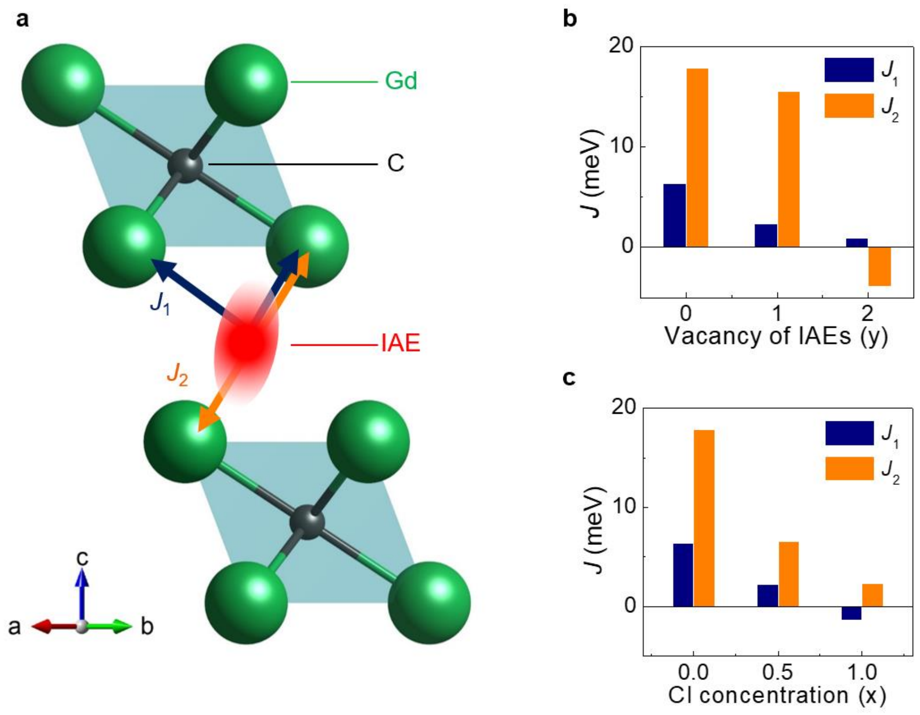

Supplementary Figure 9 Exchange interaction of the $[Gd_2C]^{2+}\cdot y\square\cdot(2-y)e^-$ and $[Gd_2C]^{2+}\cdot(1-x)2e^-\cdot Cl_x$ systems. a Schematic diagram indicating the exchange interaction between Gd atoms. J_1 is the interaction between in-plane Gd–Gd atoms, while J_2 is the interaction between out-of-plane Gd–Gd atoms across IAEs or Cl atoms. **b** Exchange interaction of $[Gd_2C]^{2+}\cdot y\square\cdot(2-y)e^-$ system. **c** Exchange interaction of $[Gd_2C]^{2+}\cdot(1-x)2e^-\cdot Cl_x$ system.

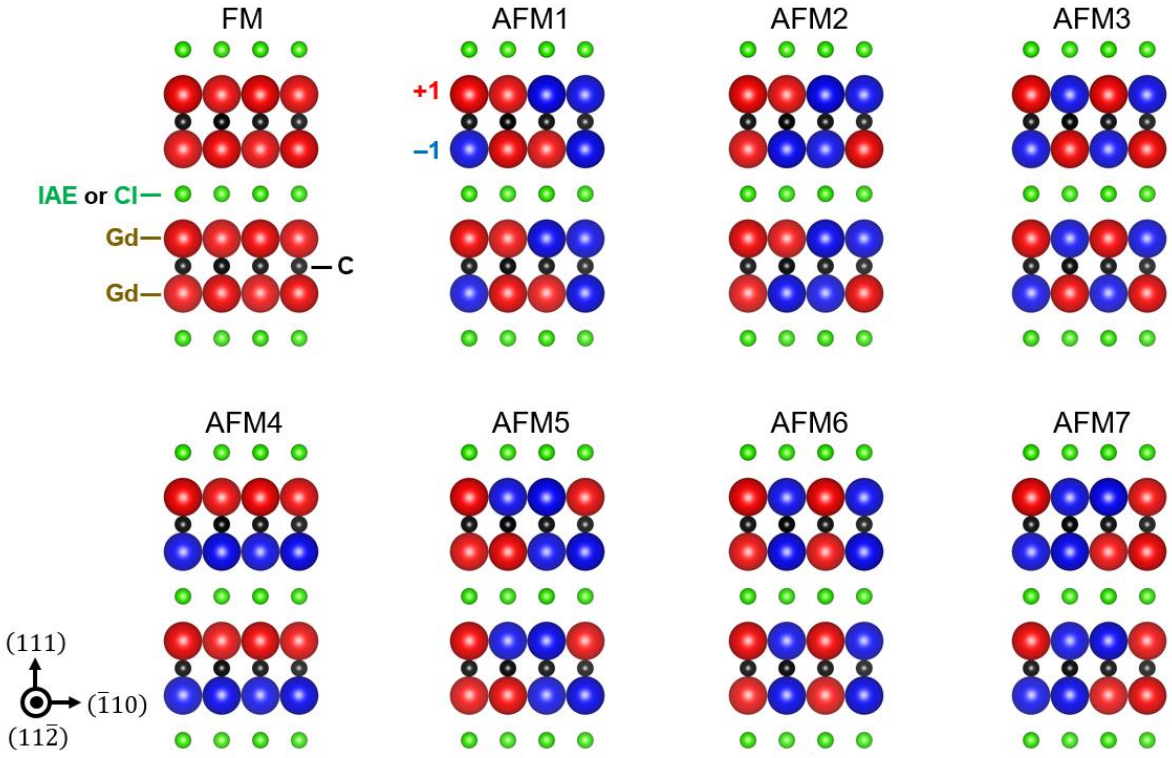

Supplementary Figure 10 Spin configurations used for the calculation of exchange interaction (*J*) in Supplementary Fig. 9 for $[Gd_2C]^{2+}\cdot(1-x)2e^{-}\cdot Cl_x$ system viewed from $(11\bar{2})$ direction. Red (Blue) spheres represent Gd atoms with the positive (negative) local magnetic moment in the *c*-axis. Green spheres represent IAEs or Cl ions while black spheres represent C atoms. Only atoms on $[11\bar{2}]$ plane are shown. With 8 Gd atoms in the unit cell used in our calculation, 8 different (1 ferromagnetic plus 7 antiferromagnetic) spin configurations are possible. Given the Heisenberg Hamiltonian for magnetic energy, $H_M = -\sum_{ij} \hat{e}_i \cdot \boldsymbol{J}_{ij} \cdot \hat{e}_j$, ($\hat{e}_i$ is the unit vector in the direction of the i-th site magnetization, J_{ij} is the exchange parameters), these eight configurations are used to determine the exchange parameters J_{ij}, which in turn determine magnetic critical temperatures T_C and T_N.

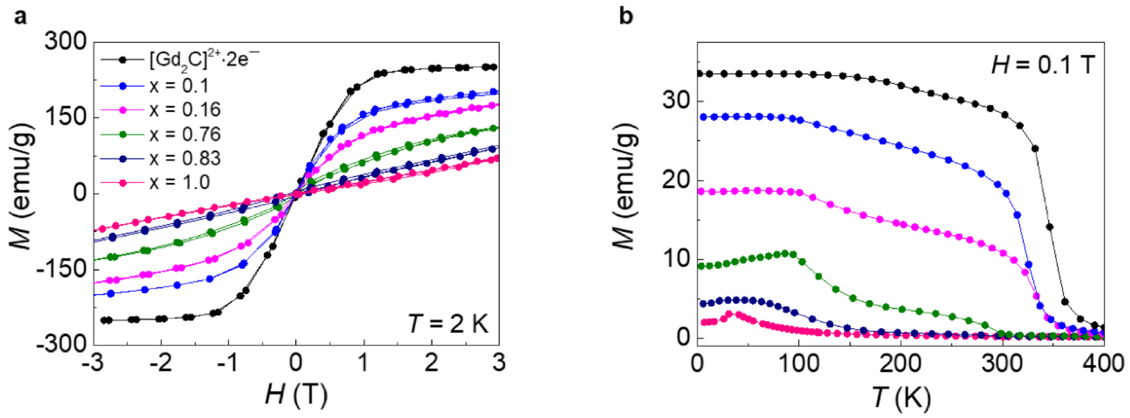

Supplementary Figure 11 Magnetic properties of $[Gd_2C]^{2+}\cdot(1-x)2e^{-}\cdot Cl_x$ system. a Magnetic field dependence of magnetization (*M*) for Cl concentration (*x*) at 2 K. **b** Temperature dependence of *M* for *x* at 0.1 T.

Supplementary Table 1 Results of Rietveld refinements for $[Gd_2C]^{2+}\cdot(1-x)2e^{-}\cdot Cl_x$ system.

System	x = 0.00	x = 0.10	x = 0.16	x = 0.76	x = 0.83	x = 1.00
Space group				$R\bar{3}m$		
Lattice parameter						
a (Å)	3.621	3.679 (6)	3.670 (5)	3.699 (1)	3.699 (2)	3.692 (1)
c (Å)	18.417	19.224 (7)	19.210 (3)	20.020 (2)	20.140 (2)	20.350 (4)
V (Å^3)	209.13	225.42	224.14	237.24	238.67	240.24
R_{wp} (%)	6.5	10.8	12.7	10.4	12.0	9.4
Atomic coordination						
Gd (0, 0, z)	0.258	0.264	0.262	0.267	0.268	0.268
C (0, 0, 0)				All identical		
e^{-} (0, 0, 0.5)				All identical		
Cl (0, 0, 0.5)				All identical (except x = 0)		
Composition						
Gd_2C	100	72.9	11.4			
$Gd_2CCl_{0.1}$		18.6		33.8	24.5	
$[Gd_2C]^{2+}\cdot(1-x)2e^{-}\cdot Cl_x$				37.1	49.9	84
$Gd_6Cl_5C_3$		8.3	27	15.3	20.5	
Gd_2Cl_2C		0.2	0.4	13.8	5.1	15.5
GdO_{2-x}						0.5

Supplementary Table 2 Electron counts and local magnetic moments of the $[Gd_2C]^{2+}\cdot(1-x)2e^{-}\cdot Cl_x$ and $[Gd_2C]^{2+}\cdot y\square\cdot(2-y)e^{-}$ systems. The quantities in parentheses are the charges transferred to each atomic site. x is the concentration of Cl atoms, while y indicates the concentration of □, which is the vacancy of the IAEs. The electron counts and local magnetic moments are computed by Bader's charge decomposition method[S3].

System	Charge (e)				Magnetic moment (μ_B)			
	Gd	C	IAE(X)	Cl	Gd	C	IAE(X)	Cl
x = 0.0	15.706 (+2.3)	6.769 (−2.8)	1.819 (−1.8)		7.46	−0.12	0.52	
x = 0.5	15.896 (+2.1)	6.978 (−3.0)	1.579 (−1.6)	7.880 (−0.9)	7.29	−0.12	0.40	−0.01
x = 1.0	15.888 (+2.1)	7.205 (−3.2)		8.018 (−1.0)	±7.11	±0.04		0.00

System	Charge (e)			Magnetic moment (μ_B)		
	Gd	C	IAE(X)	Gd	C	IAE(X)
y = 1	15.617 (+2.4)	6.708 (−2.7)	1.060 (−1.0)	7.24	−0.07	0.50
y = 2	15.621 (+2.4)	6.534 (−2.5)	0.225 (−0.2)	±7.10	0.00	0.00

Supplementary Table 3 Comparison of Bader decomposition and projection method to compute local magnetic moments. The “sum” column is computed by adding the local moments of all atoms and IAEs. The “total” column is the total magnetic moment obtained by integrating the magnetization density over the entire unit cell. While these two quantities match in the Bader decomposition method, they show a significant discrepancy in the projection method exposing the undercounting and double-counting problem inevitably present in the projection method (Supplementary Fig. 6c).

Method	Magnetic Moment (μ_B)					Vol (%)
	Gd	C	IAE(X)	Sum	Total	
Bader	7.46	−0.12	0.52	15.33	15.33	100
Projection	7.38	−0.11	0.32	14.97	15.33	92.5

Supplementary Table 4 Local magnetic moments on Gd atom and IAEs as the radii of projection spheres are changed. $\Delta r/r$ and $\Delta\mu/\mu$ represent the percentage of change in sphere radius and local magnetic moment, respectively. When the radii of projection spheres of Gd atom and IAEs are reduced by 8.3 % and 16.7 %, the local magnetic moment of Gd atom changes by a small amount (−1.22 % and −3.13 %). On the other hand, the local magnetic moment of IAEs changes significantly by −21.9 % and −40.6 %. This indicates that the local magnetic moment of the Gd atom is well localized around the nucleus and suffer little reduction when the radius of the projection sphere is reduced. On the other hand, the magnetization density of IAEs is significantly delocalized over the interstitial region and its local magnetic moment is reduced when a smaller projection sphere is employed.

	Gd			IAE		
r (Å)	1.778	1.630	1.482	1.499	1.374	1.249
$\Delta r/r$	0.0	−8.3 %	−16.7 %	0.0	−8.3 %	−16.7 %
μ (μ_B)	7.38	7.27	7.13	0.32	0.25	0.19
$\Delta\mu/\mu$	0.0	−1.22 %	−3.13 %	0.0	−21.9 %	−40.6 %

Supplementary Table 5 Results of inductively coupled plasma measurement for Gd raw material and $[Gd_2C]^{2+}\cdot 2e^-$ electride.

Ferromagnetic elements

Sample	Fe	Co	Ni	Tb	Dy	Ho	Er	Tm
**Gd**								
Sample_1	1.411	0.215	0.033	0.800	0.264	0.047	0.009	0.110
Sample_2	2.447	0.089	0.052	0.739	2.477	0.288	1.695	0.079
**$[Gd_2C]^{2+}\cdot 2e^-$**								
Sample_1	0.501	0.004	0.014	1.077	1.042	0.230	1.670	0.113
Sample_2	None	0.001	0.004	0.741	0.720	0.170	1.269	0.075
Sample_3	0.080	0.007	0.016	1.101	1.122	0.275	2.110	0.119
Sample_4	0.017	0.007	0.012	1.325	1.369	0.326	2.487	0.144
Sample_5	0.691	0.040	0.044	1.035	0.136	0.046	0.016	0.147
Sample_6	0.419	0.024	0.047	0.857	0.127	0.048	0.012	0.121

Average

	Fe	Co	Ni	Tb	Dy	Ho	Er	Tm
**Gd**	1.929	0.152	0.043	0.770	1.370	0.167	0.852	0.095
**$[Gd_2C]^{2+}\cdot 2e^-$**	0.341	0.013	0.022	1.022	0.752	0.182	1.260	0.119

Antiferromagnetic elements

Sample	Cr	Mn	Ce	Nd	Sm
**Gd**					
Sample_1	0.608	1.329	0.224	0.619	0.742
Sample_2	0.216	0.104	3.031	3.455	0.479
**$[Gd_2C]^{2+}\cdot 2e^-$**					
Sample_1	0.151	None	0.216	5.031	0.004
Sample_2	0.054	None	0.114	2.803	0.002
Sample_3	0.093	0.002	0.207	5.369	0.003
Sample_4	0.098	0.016	0.233	5.812	0.003
Sample_5	0.355	0.006	0.103	0.881	0.004
Sample_6	0.210	0.063	0.110	0.860	0.004

Average

	Cr	Mn	Ce	Nd	Sm
**Gd**	0.412	0.716	1.627	2.037	0.611
**$[Gd_2C]^{2+}\cdot 2e^-$**	0.160	0.021	0.163	3.459	0.003

Concentration [ppm]
Error range [3%]

References for Supplementary Information

S1. Lee, K. et al. Dicalcium nitride as a two-dimensional electride with an anionic electron layer. *Nature* **494,** 336-340 (2013).

S2. Park, J. et al. Strong localization of anionic electrons at interlayer for electrical and magnetic anisotropy in two-dimensional Y_2C electride. *J. Am. Chem. Soc.* **139,** 615-618 (2017).

S3. Bader, R. F. W. A quantum theory of molecular structure and its applications. *Chem. Rev.* **91,** 893-928 (1991).

S4. Bauhofer, C. et al. Struktur und eigenschaften von Gd_2XC (X=Cl,Br,I). *J. Less Common Met.* **167,** 65-79 (1990).
